# Effects of a Geriatric Assessment Intervention on Patient-Reported Symptomatic Toxicity in Older Adults With Advanced Cancer

**DOI:** 10.1200/JCO.22.00738

**Published:** 2022-11-10

**Authors:** Eva Culakova, Supriya G. Mohile, Luke Peppone, Erika Ramsdale, Mostafa Mohamed, Huiwen Xu, Megan Wells, Rachael Tylock, Jim Java, Kah Poh Loh, Allison Magnuson, Leah Jamieson, Victor Vogel, Paul R. Duberstein, Benjamin P. Chapman, William Dale, Marie Anne Flannery

**Affiliations:** ^1^ Department of Surgery, Supportive Care in Cancer, University of Rochester Medical Center, Rochester, NY; ^2^ James P Wilmot Cancer Institute, Division of Hematology/Oncology, Department of Medicine, University of Rochester Medical Center, Rochester, NY; ^3^ Department of Public Health Sciences, University of Rochester Medical Center, Rochester, NY; ^4^ School of Public and Population Health and Sealy Center on Aging, University of Texas Medical Branch, Galveston, TX; ^5^ Center for Advanced Research Technology, University of Rochester Medical Center, Rochester, NY; ^6^ Metro Minnesota Community Oncology Research Program, St Louis Park, MN; ^7^ Geisinger Cancer Institute, Danville, PA; ^8^ Department of Health Behavior, Society, and Policy, Rutgers School of Public Health, Piscataway, NJ; ^9^ Department of Psychiatry, University of Rochester Medical Center, Rochester, NY; ^10^ City of Hope National Medical Center, Department of Supportive Care Medicine, Duarte, CA; ^11^ School of Nursing, University of Rochester, Rochester, NY

## Abstract

Providing a geriatric assessment (GA) summary with management recommendations to oncologists reduces clinician-rated toxicity in older patients with advanced cancer receiving treatment. This secondary analysis of a national cluster randomized clinical trial (ClinicalTrials.gov identifier: NCT02054741) aims to assess the effects of a GA intervention on symptomatic toxicity measured by Patient-Reported Outcomes Common Terminology Criteria for Adverse Events (PRO-CTCAE).

**METHODS:**

From 2014 to 2019, the study enrolled patients age ≥ 70 years, with advanced solid tumors or lymphoma and ≥ 1 GA domain impairment, who were initiating a regimen with high prevalence of toxicity. Patients completed PRO-CTCAEs, including the severity of 24 symptoms (11 classified as core symptoms) at enrollment, 4-6 weeks, 3 months, and 6 months. Symptoms were scored as grade ≥ 2 (at least moderate) and grade ≥ 3 (severe/very severe). Symptomatic toxicity was determined by an increase in severity during treatment. A generalized estimating equation model was used to assess the effects of the GA intervention on symptomatic toxicity.

**RESULTS:**

Mean age was 77 years (range, 70-96 years), 43% were female, and 88% were White, 59% had GI or lung cancers, and 27% received prior chemotherapy. In 706 patients who provided PRO-CTCAEs at baseline, 86.1% reported at least one moderate symptom and 49.7% reported severe/very severe symptoms at regimen initiation. In 623 patients with follow-up PRO-CTCAE data, compared with usual care, fewer patients in the GA intervention arm reported grade ≥ 2 symptomatic toxicity (overall: 88.9% *v* 94.8%, *P* = .035; core symptoms: 83.4% *v* 91.7%, *P* = .001). The results for grade ≥ 3 toxicity were comparable but not significant (*P* > .05).

**CONCLUSION:**

In the presence of a high baseline symptom burden, a GA intervention for older patients with advanced cancer reduces patient-reported symptomatic toxicity.

## INTRODUCTION

More than 25% of all new cancer cases are diagnosed in patients age 75+ years.^1,2^ Older patients remain under-represented in cancer clinical trials, limiting knowledge of the safety and efficacy of treatments.^3^ Older patients with aging-related conditions (eg, disability and comorbidity) and advanced cancer experience a high prevalence of treatment-related symptomatic toxicities.^4-7^

**CONTEXT**

**Key Objective**
Can geriatric assessment (GA)–based recommendations provided to oncologists improve patient-reported symptomatic toxicities in older adults initiating a new systemic treatment regimen?
**Knowledge Generated**
In a clinical trial of older adults with advanced cancer initiating a new treatment regimen with a high prevalence of toxicity, 86% of patients reported at least one symptom of moderate or greater severity before initiating treatment. Patients who received GA recommendations had decreased symptomatic toxicities compared with usual care. However, the majority of patients in both treatment arms reported new or worsening symptomatic toxicities over 6 months following treatment initiation. The results support the use of GA recommendations in older adults with advanced cancer and reinforce the feasibility of collecting patient-reported toxicity data longitudinally from this population.
**Relevance *(S.B. Wheeler)***
GAs can inform treatment selection in older adults with advanced cancer, and high symptom burden in this population underscores the need to monitor patient-reported outcomes on an ongoing basis.*
  *Relevance section written by *JCO* Associate Editor Stephanie B. Wheeler, PhD, MPH.

Increasingly, the importance of partnering with patients to better understand symptomatic toxicity has been recognized.^4,8^ The US Food and Drug Administration has identified symptom burden and symptomatic toxicities as core concepts of interest.^9-11^ The National Cancer Institute (NCI) developed the patient-reported outcome version of the Common Terminology Criteria for Adverse Events (PRO-CTCAE) as a complement to clinician-rated CTCAE.^12-14^ The content validity,^12^ feasibility,^15,16^ reliability,^17^ and construct validity^17,18^ of PRO-CTCAE items have been established. The NCI PRO-CTCAE Library^19^ includes 78 symptom items that evaluate symptom presence/absence, frequency, severity, and interference with daily activities. Although feasibility data of PRO-CTCAE in clinical trials are growing,^15,20^ the optimal interpretation of patient-reported symptomatic toxicities is less established.^14^

Aging-related conditions influence the prevalence and reporting of symptomatic toxicities and their impact on treatment tolerability.^4,21-23^ Patients' ratings often differ from clinicians' ratings, and provide complementary but unique information on tolerance.^24-26^ In older adults with advanced cancer, it is particularly important to include the patient-reported perspective because goals of treatment often prioritize palliation of symptoms.^27^ Moreover, even mild or moderate levels of toxicities may have a negative effect on function.^28,29^ PRO-CTCAE research has included limited numbers of adults age 70+ years.^17,18,30,31^ No prior studies have incorporated PRO-CTCAEs in a randomized trial of older adults with advanced cancer and aging-related conditions.^4^

Geriatric assessment (GA) uses patient-reported and objective measures to evaluate aging-related domains.^32^ The Geriatric Assessment for Patients 70 Years and Older (GAP70+; ClinicalTrials.gov identifier: NCT02054741) cluster randomized trial demonstrated that providing a GA summary with GA-guided management recommendations to community oncologists significantly reduces serious treatment toxicity (as measured by clinician-rated CTCAE) in older patients with advanced cancer and aging-related conditions.^7^ The aims of this secondary analysis of the GAP70+ trial are (1) to evaluate patient-reported symptoms at the initiation of a new regimen and (2) to assess the effect of the GA intervention on patient-reported symptomatic toxicities as measured by PRO-CTCAE. The analysis was guided by the NCI Cancer Moonshot Cancer Treatment Tolerability consortium, which provided the opportunity to share methods and resources across investigative groups.^4,33^

## METHODS

### Study Design and Participants

This secondary analysis uses data from the GAP70+ trial, a nationwide study conducted in the University of Rochester NCI Community Oncology Research Program (UR NCORP). Community oncology practices were randomized to GA intervention or usual care. Eligible patients were age ≥ 70 years, had incurable solid tumors or lymphoma, at least one GA domain impairment other than polypharmacy, and were initiating a new systemic treatment regimen with a > 50% prevalence of grade 3-5 toxicity. Because of the high prevalence of polypharmacy in older adults^34^ and unclear association of polypharmacy with toxicity, an additional GA domain impairment was required. Patients in both arms underwent a GA^32^ before the initiation of the planned treatment regimen. In the intervention arm, oncologists were provided with GA summary and management recommendations for each enrolled patient. In the usual care arm, oncologists did not receive recommendations, but were alerted to positive screens for depression and cognitive impairment. The study was approved by the institutional review boards at all participating sites, and all patients provided informed consent. The study enrolled patients from 2014 to 2019; details on study design, participant demographic, disease, and treatment characteristics, and the effect of intervention on primary and secondary outcomes have been published previously.^7^ Briefly, along with reduced CTCAE toxicity, a greater proportion of patients in the GA intervention arm, compared with usual care, initiated treatment with a dose-reduced regimen. However, more patients in the usual care arm experienced dose reductions during treatment .^7^

### Measures

Demographics, including age, sex, ethnicity, and race, were collected as self-report measures. Cancer type and stage, history of prior chemotherapy, planned treatment, and physician-reported Karnofsky performance score^35^ were extracted from the medical record. Before the initiation of the treatment, GA domain impairments for function, objective physical performance, comorbidity, polypharmacy, cognition, social support, and psychologic status were assessed by a series of validated instruments. The GA measures are aligned with the ASCO geriatric oncology guidelines^32^ and were published previously.^7^

Symptoms were evaluated with PRO-CTCAE to assess symptomatic toxicity and provide complementary data to CTCAE. During the design of the GAP 70+ study in 2013, the investigators selected 27 PRO-CTCAE symptoms on the basis of relevance to older adults and prevalence of treatment toxicities. The items were reviewed by the NCI, geriatric oncology experts in the Cancer and Aging Research Group,^36^ patient advocates,^37^ and clinicians in the UR NCORP network. This analysis concentrates on the attribute of severity. Severity was selected since responses range from none, to mild, moderate, severe, or very severe; these responses correspond to scoring for many CTCAE items.^38^ PRO-CTCAE items with a severity attribute (24/27) are included in this analysis. Responses were scored as grade ≥ 2 (moderate or higher) or grade ≥ 3 (severe or very severe).

Core symptoms commonly occur across diseases and treatments.^39,40^ The NCI Symptom Management and Health-Related Quality of Life Steering Committee identified a set of 12 core symptoms that includes fatigue, insomnia, pain, anorexia (appetite loss), dyspnea, cognitive problems, anxiety, nausea, depression, sensory neuropathy, constipation, and diarrhea.^40^ Among PRO-CTCAE items in GAP70+, 10 core symptoms were included. PRO-CTCAE items of anxiety and depression were not included (because they were captured by GA measures). Cognitive problems were represented by two items (memory and concentration problems), so that 11 core items were collected in total. Patients completed PRO-CTCAE questionnaires on paper at baseline, 4-6 weeks, 3 months, and 6 months.

### Statistical Analysis

**FIG 1.**
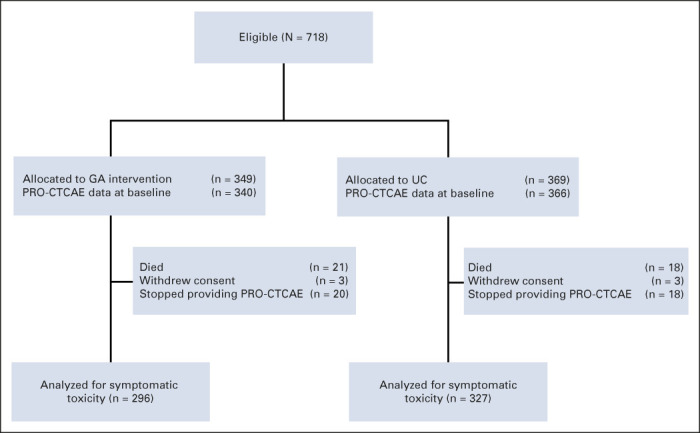
CONSORT flow diagram for symptomatic toxicity as measured by PRO-CTCAE. GA, geriatric assessment; PRO-CTCAE, Patient-Reported Outcome Version of the Common Terminology Criteria for Adverse Events; UC, usual care.

All patients who provided PRO-CTCAE data at baseline were included in the analysis of symptom burden at the initiation of the regimen, and those who also provided data for at least one postbaseline assessment were included in the analysis of symptomatic toxicity during treatment (Fig 1). For both analyses, two summary approaches were planned: (1) on the basis of all 24 severity items and (2) restricted to 11 core items.

Descriptive statistics, means, standard deviation, range for continuous measures, and n (%) for categorical measures were used to characterize the sample. To appraise baseline severity, the distribution of each symptom was visualized according to study arm. The balance in distribution of symptom severity across the two study arms was assessed using the Wilcoxon rank test. To characterize symptom burden at baseline, the overall proportion of patients who reported any symptom with moderate or higher severity were evaluated as well as proportions of patients who reported severe/very severe symptoms. The same proportions were evaluated for cores symptoms. Additionally, differences between patients who provided only baseline PRO-CTCAE data and those who provided at least one additional time point were compared by chi-square test.

To evaluate the effects of the GA intervention on the outcomes of patient-reported symptomatic toxicity, a two-step process was followed. First, following the baseline-adjusted method developed by Basch et al^41^ for each PRO-CTCAE item, the maximum severity grade reported after baseline was compared with baseline severity. For each PRO-CTCAE item, a patient was classified as having a grade ≥ 2 symptomatic toxicity event if the maximum grade reported after baseline was both grade ≥ 2 and greater than the baseline grade. Events across symptoms were combined; the binary outcome of grade ≥ 2 symptomatic toxicity was calculated as the patient experiencing a grade ≥ 2 symptomatic toxicity event for any of the 24 symptoms. The grade ≥ 2 core symptomatic toxicity was defined as patient experiencing grade ≥ 2 symptomatic toxicity event for any of the 11 core items. The definition of grade ≥ 3 symptomatic toxicity followed an analogous process. Both grade ≥ 2 and grade ≥ 3 symptomatic toxicity outcomes were prespecified before the analysis.

Between-arm differences in symptomatic toxicities were compared using the chi-square test. To account for correlations between patients from the same practice cluster, we further assessed the effect of the intervention on symptomatic toxicity using a generalized estimating equation^42^ regression model with the binary outcome and log link. The adjusted risk ratio (ARR) from the generalized estimating equation model together with 95% robust CI is reported. To better understand the contribution of individual PRO-CTCAE items, the between-arm differences for each symptom were assessed using the same analytical approach. Sensitivity analyses assessing the effect of the intervention on symptomatic toxicity in prespecified subgroups by cancer type, treatment type, and history were also performed. Statistical significance was set at a two-sided alpha = .05 level. The findings reported in this manuscript should be considered as hypothesis-generating. Data were analyzed using SAS version 9.4 (SAS Inc, Cory, NC).

## RESULTS

### Patient Characteristics

Of 718 patients enrolled onto GAP70+, 706 (98.3%) provided PRO-CTCAEs at baseline. Of 706 patients, 83 (11.8%) patients provided PRO-CTCAE data only at baseline and could not be included in the analyses of symptomatic toxicity. Of these 83 patients, 39 (47.0%) died within 4-6 weeks, six (7.2%) withdrew from the study, and 38 (45.8%) declined or were not further able to complete PRO measures (Fig 1). The death of nine patients was classified as toxicity-related (five in usual care and four in GA intervention arm). Proportions of patients reporting PRO-CTCAE data at each time point by arm were similar (Appendix Table A1, online only). The average age of the 706 patients was 77 years (standard deviation = 5.4, range, 70-96 years), 305 (43.2%) were female, and 182 (26.5%) had prior chemotherapy (Table 1). Patients with GI cancers (*P* = .043), lower performance status (Karnofsky performance score ≤ 80, *P* = .002), and those who had GA domain impairments in nutrition (*P* = .002), psychologic status (*P* = .002), and cognitive status (*P* = .048) were more likely to not provide PRO-CTCAE data after baseline (Table 1).

**TABLE 1.**
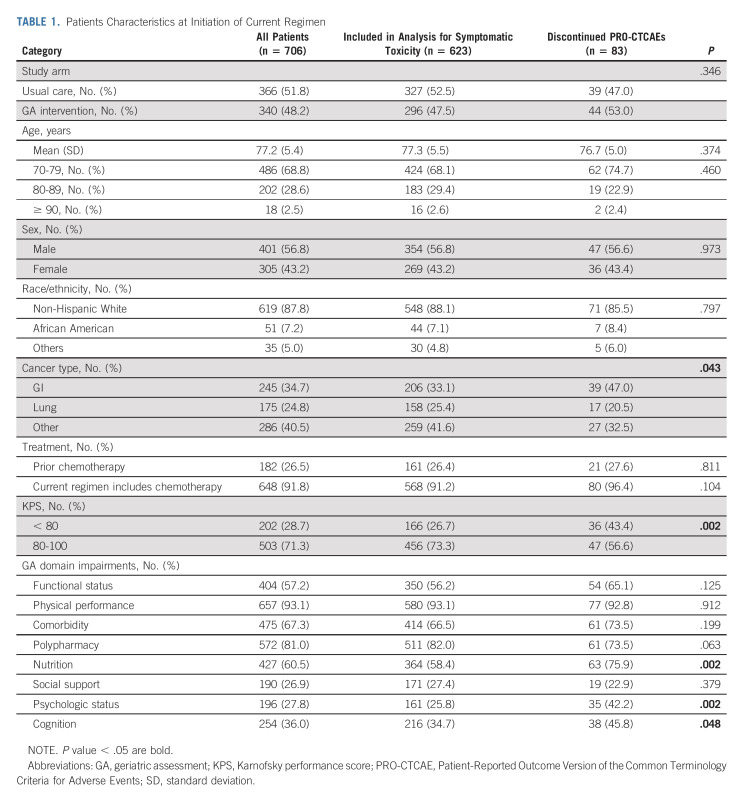
Patients Characteristics at Initiation of Current Regimen

### Baseline Symptom Burden as Measured by PRO-CTCAE

**FIG 2.**
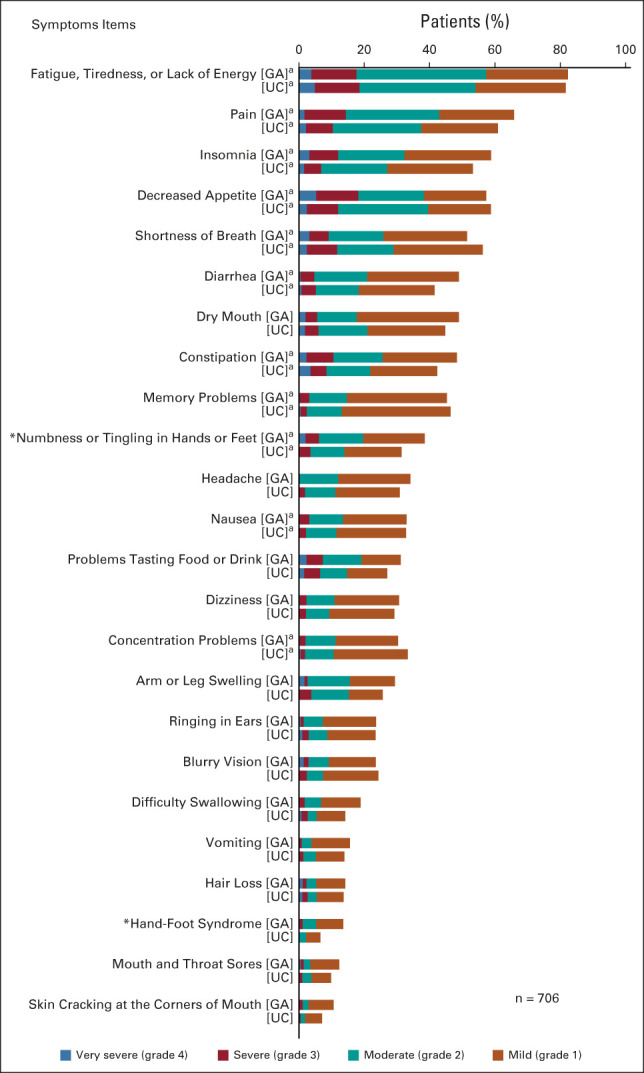
Baseline symptom severity by arm. ^a^Core symptom. *P < .05. GA, geriatric assessment intervention; UC, usual care.

**FIG 3.**
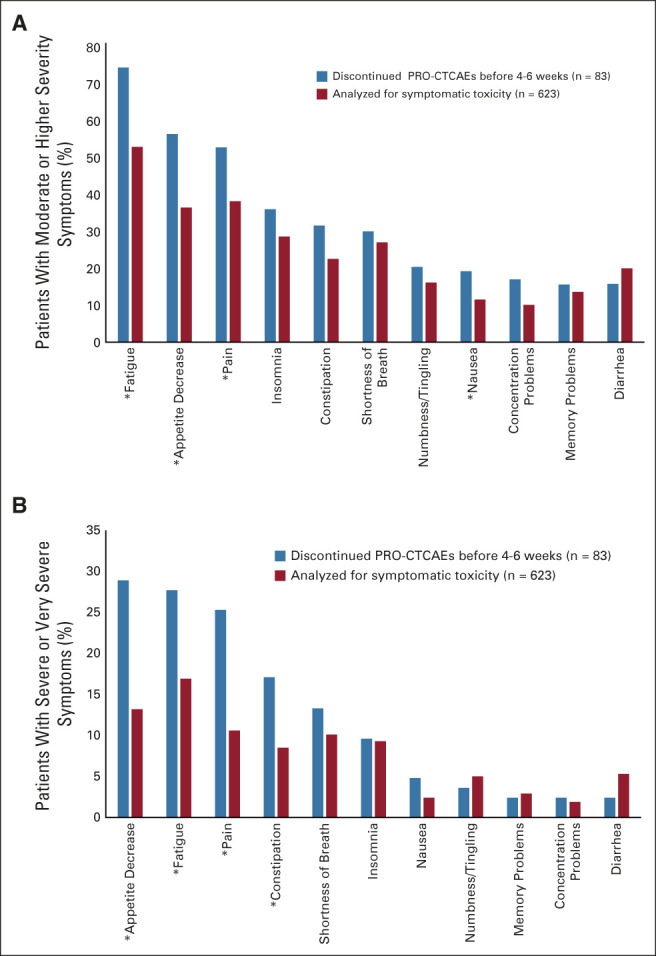
Patients reporting of symptom severity at baseline: (A) moderate or higher severity and (B) severe and very severe. *P < .05. PRO-CTCAE, Patient-Reported Outcome Version of the Common Terminology Criteria for Adverse Events.

**FIG A1.**
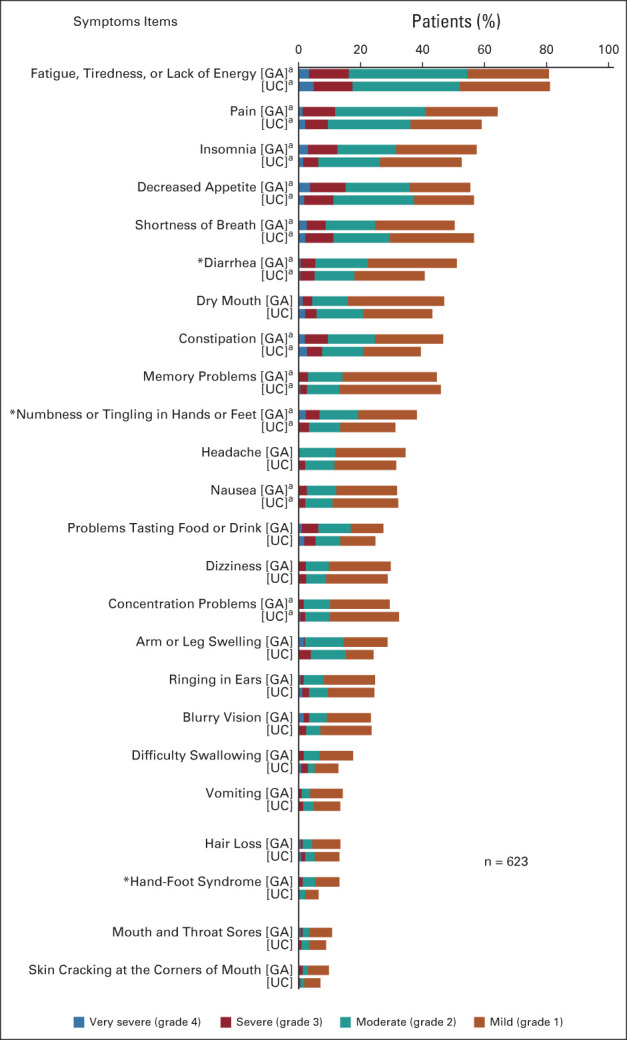
Baseline symptom severity by study arm in patients analyzed for symptomatic toxicity. ^a^Core symptom. *P < .05. GA, geriatric assessment intervention; UC, usual care.

Figure 2 presents the distribution of severity of symptoms by study arm before treatment initiation. The baseline distribution was well balanced between the arms, with a statistically significant difference detected only for numbness/tingling (*P* = .027) and hand-foot syndrome (*P* = .002). The most prevalent symptoms were fatigue (82.0%), pain (63.3%), and decreased appetite (58.2%). The six most prevalent symptoms were core symptoms (Fig 2, Appendix Fig A1, online only). Of 706 patients, 608 (86.1%) reported at least one symptom with moderate or higher severity, and 579 (82.0%) reported at least one core symptom with moderate or higher severity. Almost half (351; 49.7%) reported at least one severe or very severe symptom, and 310 (43.9%) reported at least one core symptom as severe/very severe (Appendix Table A2, online only). The proportions did not significantly differ by study arm. Patients who provided only baseline PRO-CTCAE (n = 83) were more likely to report a core symptom with moderate or higher severity (92.8% *v* 80.6%, *P* = .007) and severe or very severe core symptom (67.5% *v* 47.4%, *P* = .001). Specifically, they reported a higher prevalence of moderate or greater severity for fatigue, pain, and decreased appetite (Figs 3A and 3B), and of severe/very severe constipation (all *P* < .05; Fig 3B). The proportions reporting moderate or higher severity at baseline did not differ significantly by arm in 706 patients who had baseline PRO-CTCAE data or in 623 patients who provided data analyzed for symptomatic toxicity (Appendix Table A3, online only).

### Symptomatic Toxicity

**FIG 4.**
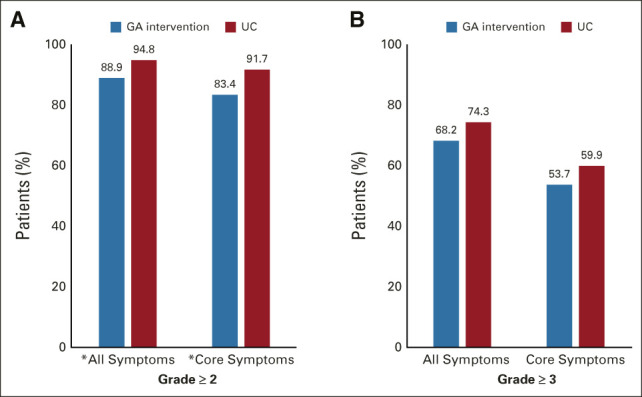
Effect of the GA intervention on patient-reported symptomatic toxicity reported over 6 months: (A) grade ≥ 2 and (B) grade ≥ 3. *P < .05. GA, geriatric assessment; UC, usual care.

**FIG A2.**
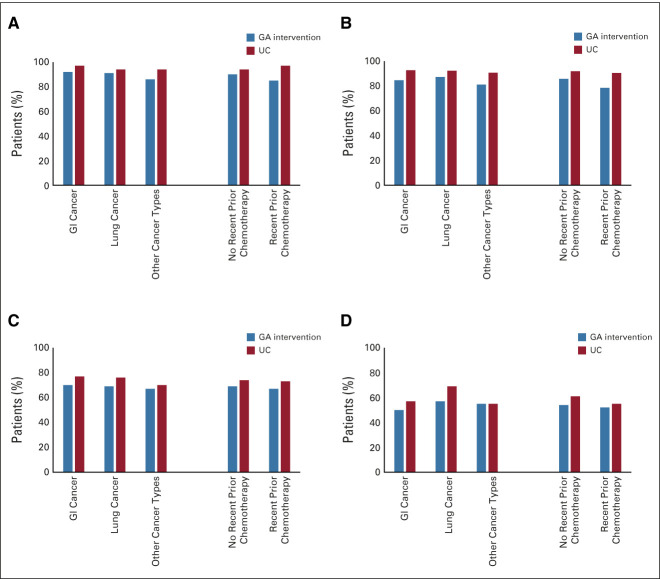
Patient-reported symptomatic toxicity over 6 months stratified by cancer type and prior treatment history: (A) grade ≥ 2 (all symptoms), (B) grade ≥ 2 (core symptoms), (C) grade ≥ 3 (all symptoms), and (D) grade ≥ 3 (core symptoms). GA, geriatric assessment intervention; UC, usual care.

After baseline, compared with usual care (n = 327), a lower proportion of patients who received the GA intervention (n = 296) reported grade ≥ 2 symptomatic toxicity (88.9% *v* 94.8%; ARR = 0.937, 95% CI, 0.882 to 0.996; *P* = .035). The proportion of grade ≥ 2 core symptomatic toxicity was also lower in the GA intervention arm compared with usual care (83.4% *v* 91.7%; ARR = 0.907, 95% CI, 0.860 to 0.962; *P* = .001; Fig 4). A similar pattern was observed for grade ≥ 3 toxicities; however, the results did not reach statistical significance (Fig 4). In stratified analysis by cancer type and prior chemotherapy, the overall pattern of higher severity of symptomatic toxicity in the usual care arm persisted (Appendix Fig A2, online only).

When evaluating individual toxicities, a lower proportion of patients who received the GA intervention reported grade ≥ 2 symptomatic toxicity on all core symptoms except for decreased appetite (Table 2).

**TABLE 2.**
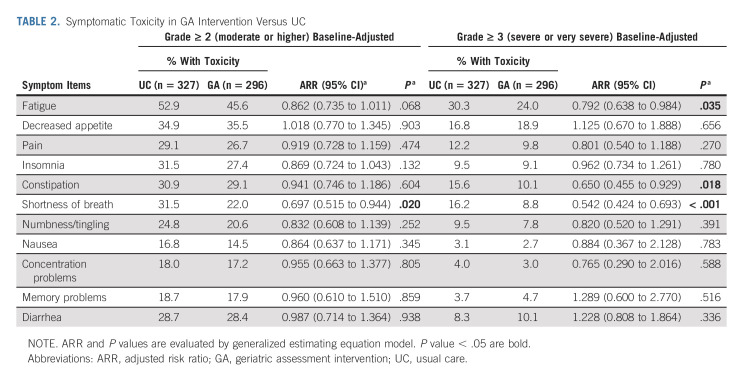
Symptomatic Toxicity in GA Intervention Versus UC

## DISCUSSION

These analyses address important gaps in understanding the symptom experience of older adults with advanced cancer receiving systemic therapy. First, Gap70+ is the first nationwide cluster-randomized trial to demonstrate that a GA intervention can decrease patient-reported symptomatic toxicities. Second, the findings establish high baseline symptom burden and a high prevalence of developing new or worsening symptomatic toxicities over 6 months. Third, to our knowledge, it is the first study to systematically describe baseline symptom burden as measured by PRO-CTCAE in older adults with advanced cancer. Fourth, this study is one of a very few to report patient-reported symptomatic toxicities as an outcome in a randomized controlled trial. Previously, PRO-CTCAEs were evaluated in trials examining the efficacy of therapeutic agents in advanced prostate cancer and non–small-cell lung cancer.^26,43^ Last, the results reinforce the feasibility of collecting PRO-CTCAE longitudinally from older adults with advanced cancer and aging-related conditions.

These results provide further support for GA-based models of care.^32^ A significant decrease in the proportion of patients reporting the development of grade ≥ 2 symptomatic toxicity over 6 months was found for patients receiving the GA intervention. Older individuals may have a decreased ability to tolerate even low-grade symptomatic toxicities because of concurrent functional decline and competing comorbidities.^28,29^ The overall effect on symptomatic toxicity was smaller than the effect found for CTCAE^7^ and was significant for grade 2 but not grade 3. This may be because GA intervention was administered at baseline only and did not specifically address symptoms. Furthermore, the sample size may not provide sufficient power to establish statistical significance for symptomatic grade ≥ 3 toxicity. A smaller difference for patient-reported PRO-CTCAE compared with clinician-rated CTCAE is consistent with the complementary purpose of these measurement systems.^44^ Prior reports indicate that patient-reported symptoms are higher in prevalence and greater in severity than clinician-rated, which may explain the smaller difference found for PRO-CTCAE results.^26,45,46^

Symptomatic toxicities were highly prevalent in both arms over 6 months. At baseline, patients had a high prevalence (86%) of having at least one moderate to severe symptom, and also the majority (92%) experienced worsening or new symptomatic toxicities. Given that an important treatment goal in the advanced cancer setting is to decrease tumor burden and improve symptom control,^47^ it is troubling that the symptomatic toxicity was so prevalent. This result reinforces the need to integrate guideline-supported palliative care^48^ with GA-informed care; the preliminary efficacy of one integrated model was recently reported in a promising pilot trial.^49,50^ In the GAP70+ trial, although oncologists reduced treatment intensity at cycle 1 and provided management for aging-related conditions, there was no systematic provision of symptom management recommendations.^7^ Emerging consensus on the efficacy of routine electronic symptom reporting and follow-up interventions could also strengthen symptom reduction in this population.^20,51-53^ The efficacy of weekly electronic PRO monitoring for adults with metastatic cancer receiving treatment (including symptoms with alerts to clinicians for severe or worsening symptoms) on improving symptoms has recently been reported.^54^ A similar symptom-specific intervention that integrates GA could be evaluated for older patients with aging-related conditions. Further research is needed to assess the feasibility of digital methods for capture of PRO data from older adults with advanced cancer and aging-related conditions.

The specific symptoms of fatigue, pain, insomnia, decreased appetite, and dyspnea were reported by more than 50% of older adults before treatment initiation. These results align with past research on commonly experienced symptom prevalence in individuals of all ages with advanced cancer.^55-57^ In GAP70+, older adults with advanced cancer who had the highest baseline symptom burden more often did not complete PRO-CTCAE at follow-up. This result is consistent with other research that has found individuals who are ill are less likely to complete PRO measures.^15,16^ It is important to offer assistance to older adults who need help with completing PRO measures.^21^ Other studies have evaluated the benefit of capturing symptom data from older adults using different PRO questionnaires. For example, Battisti et al^58^ found that the symptoms increased on the European Organisation for Research and Treatment of Cancer Quality-of-Life Questionnaires (EORTC-QLQ) C30 in older patients with early breast cancer receiving chemotherapy. Symptom assessment instruments need to be selected for clinical trials on the basis of specific study aims, ease of use in setting, and interpretation. For the GAP 70+ study, PRO-CTCAE was included because it was designed as a complement to clinician-rated CTCAE.

Ongoing efforts are underway to determine best approaches for analyzing and reporting PRO-CTCAE data.^59,60^ An aggregate measure, summing data for PRO-CTCAE items, was used. This summary measure parallels reporting for CTCAE by clinicians in clinical trials.^7^ The baseline-adjusted method was used to estimate symptomatic toxicity,^41^ and although these methods were successfully implemented^26,33^ for this analysis, methodologic challenges remain. The method does not capture toxicity for patients entering treatment with the highest symptom severity scores. Thus, the method may underestimate symptomatic toxicity for symptoms that are severe and highly prevalent at baseline.^26^ Also unique in this examination is a focus on core symptoms as recommended by the NCI.^40^

There were several study limitations. The sample was primarily White, limiting generalizability. The selected time intervals were a limitation since the PRO-CTAE items were not collected weekly, and thus may not have captured the period when symptomatic toxicities were present or most severe.^61^ The study was not fully powered for the outcome of symptomatic toxicity. Causal attribution of symptoms to treatment alone is particularly challenging in older patients with a high prevalence of aging-related conditions. Even with these limitations, this study adds to growing evidence that PRO-CTCAEs are feasible for patients to report symptoms during treatment. This analysis demonstrates their value for older patients with advanced cancer and aging-related conditions being cared for in community oncology clinics.

This analysis provides evidence that a GA intervention can decrease the prevalence of symptomatic toxicities as measured by patient-reported outcomes. Future trials should examine whether GA-based models of care that integrate symptom monitoring and management can further improve outcomes of older patients with advanced cancer and aging-related conditions.^49^

## Figures and Tables

**TABLE A1.**
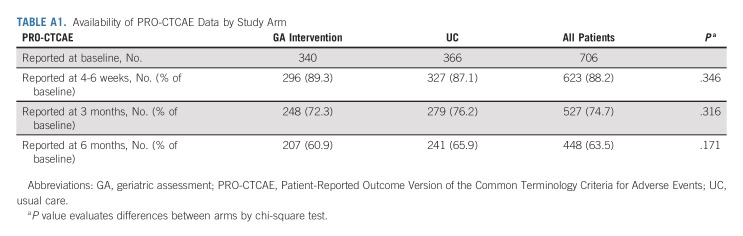
Availability of PRO-CTCAE Data by Study Arm

**TABLE A2.**
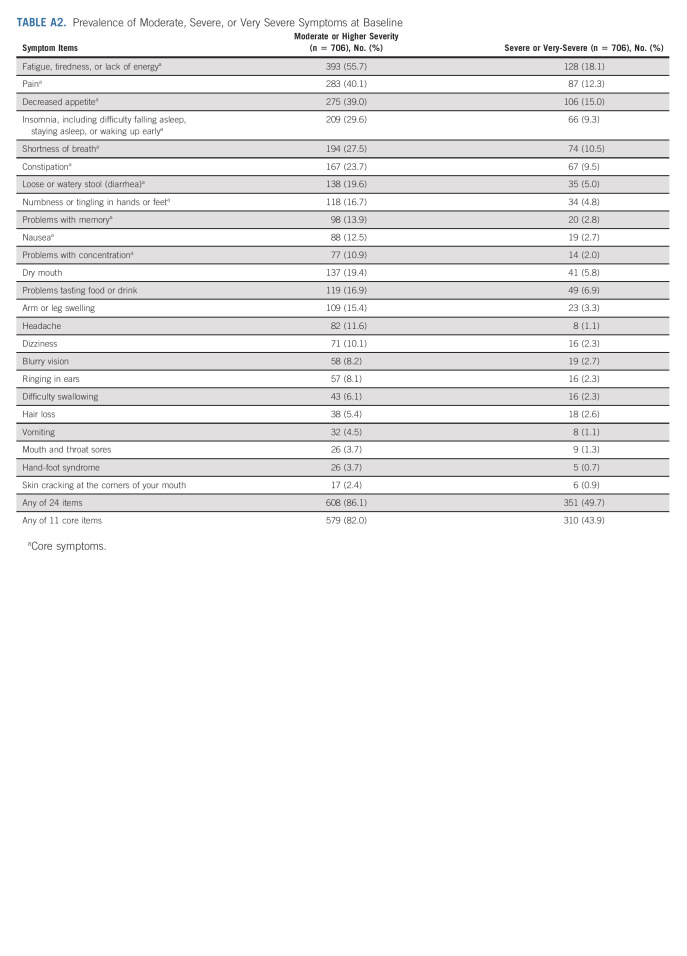
Prevalence of Moderate, Severe, or Very Severe Symptoms at Baseline

**TABLE A3.**
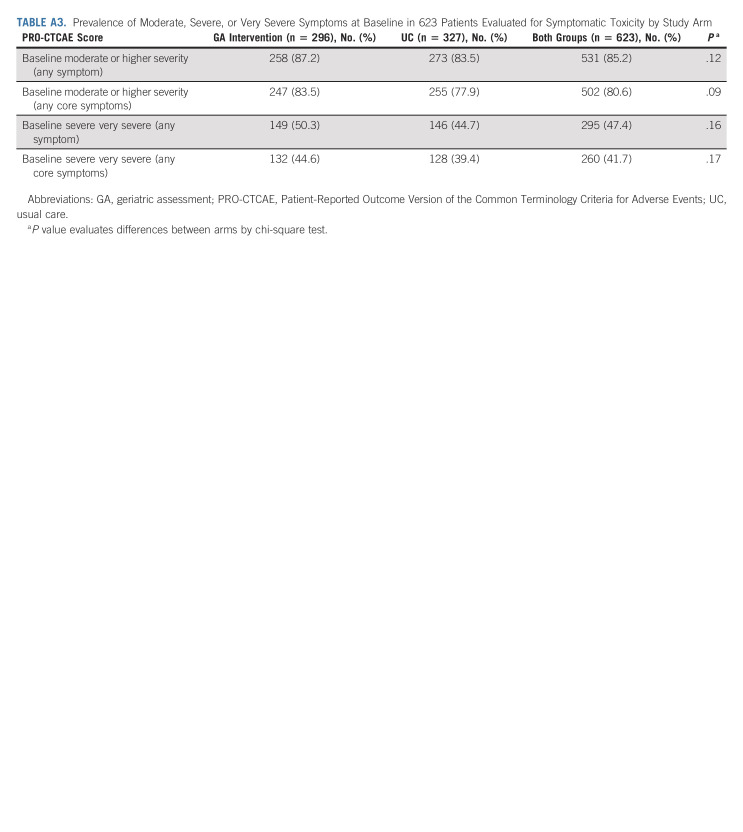
Prevalence of Moderate, Severe, or Very Severe Symptoms at Baseline in 623 Patients Evaluated for Symptomatic Toxicity by Study Arm

## Data Availability

The study protocol, statistical analysis plan, informed consent form, and clinical study reports are available on the Cancer and Aging Research Group website (https://www.mycarg.org/). These documents will be available beginning 6 months and with no end date following publication of the article. The above data and materials are made available to anyone who wishes to use the data. For any further data or materials, research proposals can be directed to SM (supriya_mohile@urmc.rochester.edu). Opportunities for further analyses will be made available to investigators of the Cancer and Aging Research Group. There is no cost to be a member of the Cancer and Aging Research Group (see https://www.mycarg.org/ for membership information).
